# Wrist-ankle acupuncture alleviates pain in the acute phase of herpes zoster: A randomized controlled trial

**DOI:** 10.1371/journal.pone.0318386

**Published:** 2025-05-29

**Authors:** Jing Pu, Daiwen Li, Xia Luo, Juan Wang, Yanxia Li, Li Lei, Xiankun Zhao, Huan Du, Xiyue Yang, Xiaobo Du

**Affiliations:** 1 Dermatological department, Mianyang Central Hospital, Mianyang, PR China; 2 Department of medicine, University of Electronic Science and Technology of China, Chengdu, PR China; 3 Department of oncology, Mianyang Central Hospital, Mianyang, PR China; 4 Sichuan Clinical Research Center for Radiation and Therapy, Mianyang, China; China Medical University, TAIWAN

## Abstract

Alternative therapeutic strategies for herpes zoster, especially for acute phase pain relief, are still largely unexplored. This study aimed to compare the effects of wrist-ankle acupuncture combined with standard pharmacological treatment versus standard pharmacological treatment alone in relieving pain in the acute phase of herpes zoster. An open-label, randomized, controlled clinical trial was conducted, enrolling patients diagnosed with acute-phase herpes zoster with pain visual analog scale (VAS) scores greater than or equal to 2. The participants were randomly assigned to either the control group receiving standard pharmacological treatment (antiviral therapy combined with pain relievers) alone or the experimental group receiving wrist-ankle acupuncture plus standard pharmacological treatment. VAS pain scores were recorded on days 1–7 and on day 28 after treatment began. Dermatology Life Quality Index scores were assessed both during the pre-treatment phase and at hospital discharge. A total of 106 patients completed the trial protocol and were included in the analysis, with 52 and 54 patients in the control and experimental groups, respectively. The clinical cure rates of pain (the rate of complete absence of pain) in the experimental group was statistically higher than control group on days 7 after treatment began(87.04% vs 65.38%, p < 0.005). The average pain VAS scores of the experimental group were lower than the control group on days 2–6 after treatment began, and they are statistically significant (all p < 0.05). No significant difference was observed between day 7 and day 28 after treatment began (*p* > 0.05). The Dermatology Life Quality Index scores significantly differed at hospital discharge (*p* < 0.05). Side effects did not significantly differ between the two groups (all *p* > 0.05). Wrist-ankle acupuncture combined with standard pharmacological treatment may potentially improve the pain cure rate at 7 days post-treatment. This suggests a potential new strategy for alleviating pain in patients in the acute phase of herpes zoster. **Trial registration:** ChiCTR2300071795.

## Introduction

Herpes zoster (HZ) is caused by the varicella-zoster virus (VZV) reactivation in previously infected individuals. It manifests as a band-like rash in the dermatome corresponding to the affected nerve [1]. The lifetime risk of herpes zoster is 25–30%, and the risk increases to 50% for those aged 85 years and above [2, 3]. Predominantly, individuals with HZ experience burning or stinging pain, affecting approximately 90% of patients [4]. Herpes zoster-associated pain (ZAP) is divided into three phases: (1) acute herpes zoster pain, mainly represents the acute rash convalescence; (2) subacute herpetic neuralgia, typically persisting beyond the acute phase without developing into chronic pain; and (3) postherpetic neuralgia (PHN), is defined as persistent pain lasting 120 days or more after rash onset [5].

The antiviral treatment duration for HZ is generally 7 days and can be extended to 10–14 days under specific circumstances. While antiviral drugs are essential for the treatment of HZ, they do not provide sufficient pain reduction during the acute phase [6, 7]. Consequently, pain relievers drugs are commonly prescribed with antivirals to alleviate pain in the acute phase [8]. However, combination therapy may increase the incidence of nephrotoxicity [9]. Moreover, the considerable side effect profiles of the commonly used oral medications often limit their practical use, and a combination of both topical and systemic agents may be required for optimal outcomes. [8]. Alternative therapeutic strategies for HZ, especially for acute phase pain relief, remain largely unexplored.

Chinese medicine, particularly Traditional Chinese Medicine and acupuncture, has been used to alleviate ZAP [10–12]. Acupuncture, known for its ease of implementation and minimal side effects, has been used to relieve various types of pain, including HZ pain [13, 14–18]. At present, some protocol of reviews and randomized controlled studies have begun to evaluated the effect of acupuncture on relieving acute HZ pain, but the results have not been reported yet[19,20].

Wrist-ankle acupuncture (WAA) is a therapy that involves regional acupuncture on the wrist and ankle to treat systemic diseases. It is characterized by its simplicity, safety, effectiveness, and absence of severe adverse side effects [21]. WAA has been successfully employed to alleviate various types of pain with satisfactory results [21–25]. In dermatology, the total effective rates of WAA therapy were reported to be 90.88%. However, the sample size of these studies was small, and the treatment was mainly used to alleviate PHN in HZ.

It remains uncertain whether combining acupuncture with standard pharmacological treatment offers superior pain relief during the acute phase of HZ compared to standard pharmacological treatment alone.

The purpose of this study was to compare the effects of combining WAA with standard pharmacological treatment versus standard pharmacological treatments alone in relieving pain in the acute phase of HZ.

## Methods

### Sample size calculation

The sample size was calculated using Pass software, considering two independent samples from randomized controlled trials, with a significance level of 5% and power of 80%. According to the literature, the clinical cure rate of pain on 7 days after treatment began with standard pharmacological treatment (antiviral therapy combined with pain relievers drugs) was reported to be 60%[10]. According to another literature, compared cure rates of standard medication, the relative risk combination of novel interventions (Chinese herbal medicine, acupuncture, moxibustion, or cupping) was 1.4, which exhibited better cure rates of pain symptoms in patients with acute Herpes Zoster [11]. We estimated that the clinical cure rate of pain on 7 days after treatment began in the WAA plus standard pharmacological treatment group will be 85%. To achieve this, it was necessary to recruit 96 patients. Assuming a 10% attrition rate, a total of 106 patients would be required.

### Study protocol

This study was an open-label, randomized, controlled clinical trial conducted at Mianyang Central Hospital. Inclusion criteria encompassed: 1) inpatients diagnosed with herpes zoster; 2) aged 18–80 years old; 3) pain visual analog scale (VAS) score of 2 or higher; 4) disease duration of 1–7 days with no prior treatment; 5) willingness to accept treatment; 6) signed informed consent form. Exclusion criteria included the following: 1) special types of HZ (Ocular and aural HZ, visceral, meningitis, generalized, and rash-free HZ); 2) cardiovascular or cerebrovascular disease, liver or kidney damage, thrombocytopenia, coagulation dysfunction, malignant tumors, and other diseases; 3) pregnant or lactating women. Enrolled patients met all the inclusion criteria and were randomized into control and experimental groups by the research team using computer-generated randomization. The control group received standard pharmacological treatment, while the experimental group received WAA plus standard pharmacological treatment. Patients received VAS pain scores on days 1–7 and day 28 after treatment began, and Dermatology Life Quality Index (DLQI) scores were assessed pre-treatment and at hospital discharge. VAS pain scores and DLQI will evaluated by independent nurses, who are blinded to the grouping of patients. This study was approved by the Ethics Committee of Mianyang Central Hospital (Number: S20230205-02, approval date: 2023-04-14). The study was supervised and managed by the ethics committee. This study was registered in the Chinese Clinical Trial Registry (number: ChiCTR2300071795, registration date:2023-05-25).

### Goal of the study

The primary endpoint of the study was the clinical cure rate of pain in the two groups on 7 days after treatment began. The secondary endpoints were **VAS pain scores** on day 1–7, 28 after treatment began, the change in DLQI score for the two groups from pre-treatment to hospital discharge and safety.

### The intervention

The control group received standard pharmacological treatment, which comprised antiviral therapy combined with pain relievers drugs. Specifically, the antiviral therapy used was acyclovir (3 × 5–10 mg/kg/d KG I.V. or 5x800 mg/d P.O.). Pain relievers drugs included pregabalin (75 mg/q12h P.O.), lofencodeine (26 mg/q12h P.O.), aminophenol tramadol (37.5 mg/q8h P.O.), or a combination of two of the above three drugs.

The experimental group received standard pharmacological treatment along with WAA for 30 minutes daily, administered continuously for 7 days. Different wrist and ankle positions were selected for acupuncture according to the different positions of HZ lesions [26]. The sides of the body and each limb were divided longitudinally into 6 areas, each defining an acupuncture point on the wrist or ankle. Needling acupoints can alleviate pain in the corresponding areas. Specifically, if the pain is localized in the upper body (bounded by the diaphragm, the diaphragm in traditional Chinese medicine is a line located below the spinous process of the 7th thoracic vertebra, rather than the diaphragm dissected by Western medicine.), acupuncture needles are inserted into acupoints on the same side of the wrist. Conversely, if the pain is in the lower body, acupoints on the same side of the ankle are targeted for needle insertion [26, 27].

Before commencing the intervention, several preparatory steps were undertaken. First, the selected area for the acupuncture was carefully determined, avoiding local blood vessels. Additionally, the acupuncture points and the position of the insertion point were adjusted appropriately based on the actual situation of the skin, including the presence of any scars.

Using an acupuncture (Shukang brand 0.25 mm × 25 mm milli needle, Changchun Aikang Medical Equipment limited company, Changchun, China), the insertion point was selected as the center. The skin around the insertion point was disinfected with 75% alcohol twice. Subsequently, the wrist-ankle acupuncture was inserted into the subcutaneous area at a 30° angle. After confirming that the needle tip had penetrated the dermis, the needle handle was gently twisted to slowly insert the needle along the superficial position of the subcutaneous area until the needle body was 2 mm exposed. The needle handle was then fixed with tape.

During acupuncture, it is advisable to feel loose without resistance, and the patient should not have any feelings such as soreness, numbness, swelling, or pain[26]. If discomfort such as soreness, numbness, swelling, or pain occurs during the puncture process, the acupuncture insertion angle should be adjusted based on the feedback of the patient, and the needle should be re-inserted and fixed. We have two acupuncturists to treat the patients. They have been working for more than 5 years and trained by the research group. They give acupuncture to patients in strict accordance with the research protocol, which can ensure the consistency of patients’ treatment.

If someone in the control group or experimental group no longer reports pain, their standard pharmacological treatment will be stop, and WAA treatment will also stop in the experimental group. After receiving 7 days of WAA treatment, patients in the experimental group will stop receiving WAA treatment even if someone no longer reports reduced pain, but will continue to receive standard pharmacological treatment for hospitalized patients. When patients leave hospital, if their pain score does not drop to 0, they will continue to receive outpatient medication treatment but not WAA treatment.

### Assessment of pain and DLQI

Pain was assessed using the VAS. According to the pain VAS evaluation method, the most painful point was recorded within 24 hours before the observation point (0: painless; 1–3: mild pain; 4–6: tolerable, denoting pain that interferes with sleep; 7–10: Severe, denoting pain that is intense, unbearable, affects appetite and sleep). Pain assessment occurred on days 1–7 and day 28 after treatment began unless pain completely subsided. Treatment effects on pain included clinical cure (pain disappearance), significant effect (two points reduction in pain intensity), Effective (one point reduction in pain intensity), and Invalid (less than one point reduction in pain intensity). Clinical cure defined as a complete absence of pain, clinical cure rate defined as the number of cured/total number*100%[11].

The DLQI questionnaires were self-administered, accessible, and user-friendly [28]. They comprised 10 questions concerning the perception of the patient concerning the impact of skin diseases on different aspects of their Quality of Life (QoL) over the past week. The questions were classified into 6 headings: symptoms and feelings (questions 1 and 2), daily activities (questions 3 and 4), leisure (questions 5 and 6), and personal relationships (questions 8 and 9), with a maximum score of 6 for each item in these groups. Work and school (question 7) and treatment (question 10), with a maximum score of 3 for each item. The total score ranged from 0–30, with a higher score indicating a more significant impairment of the QoL of the patient. A score of 0 indicates that the disease does not affect the QoL of the patient, while a score of 30 indicates maximum impairment of QoL.

### Safety assessment

Every patient participating in this study underwent a safety assessment, including evaluating the side effects of the medication and WWA. These assessments encompassed monitoring for nephrotoxicity, hepatotoxicity, gastrointestinal disorders, fainting associated with acupuncture, needle breaks, and hematoma.

### Statistical analysis

Our statistical analysis was conducted by two independent researchers who did not participate in patient treatment and were unaware of patient grouping. The Kolmogorov-Smirnov test was employed to assess the normality of continuous variables. Normally distributed variables were expressed as mean ± standard deviation and compared using the independent samples t-test, while non-normally distributed variables were expressed as medians and interquartile ranges and were compared using the Mann-Whitney U test. Categorical variables were presented as counts and percentages, and comparisons were performed using Pearson’s chi-square or Fisher’s exact test. A p-value < 0.05 based on a two-tailed test was considered statistically significant. All statistical analyses were performed using Statistical Package for the Social Sciences (SPSS) software (version 22.0; SPSS Inc., Chicago, Illinois, USA).

## Results

### Clinical characteristics

**Fig 1:**
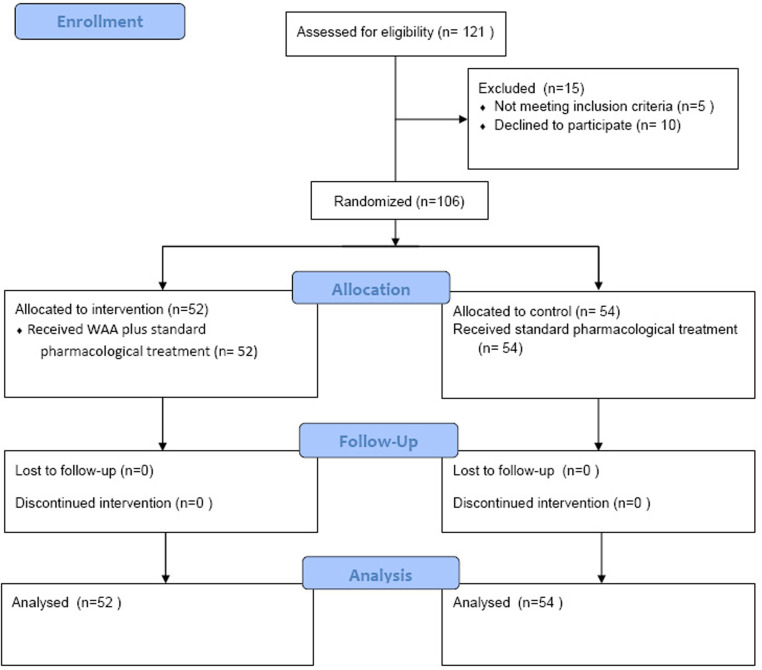
Study scheme. After screening 121 patients, a total of 106 of them were randomized. Participants who met inclusion criteria were randomized to control group or experimental group. The control group received standard pharmacological treatment, while the experimental group received WAA plus standard pharmacological treatment. Finally, 52 patients in control group and 54 patients in experimental group were analyzed for the results.

The trial was conducted from initial registration in May 2023 to the final follow-up of the last patient in March 2024. A total of 106 patients diagnosed with HZ were enrolled in this study (Fig 1). Table 1 presents the clinical characteristics of the patients.

**Table 1:** Clinical characteristics.

Clinical characteristics	Total (N = 106)	Control Group (N = 52)	Experimental Group (N = 54)	*p*-value
Age in years, mean ±SD	61.99 ± 12.34	62.21 ± 13.55	61.78 ± 11.19	0.857
Gender, n (%)				0.503
Female	70(66.04)	36(69.23)	34(62.96)	
Male	36(33.96)	16(30.77)	20(37.03)	
VAS pain score of pre-treatment, median (IQR)	5.00( 1.00)	5.00(1.00)	5.50(1.00)	0.434
DLQI score of pre-treatment, median (IQR)	15.50(2.25)	15.00(2.00)	16.00(3.00)	0.568
Hospitalization time in days, median (IQR)	8.00(2.00)	8.00(2.00)	8.00(2.0)	0.552

VAS: Visual Simulation Scale; DLQI: Dermatology Life Quality Index; SD: standard deviation; IQR: interquartile range

*The age, VAS pain scores and DLQI score were compared using the Mann-Whitney U test, the gender was compared using the Pearson’s chi-square test.

### VAS pain scores between the two groups

**Fig 2:**
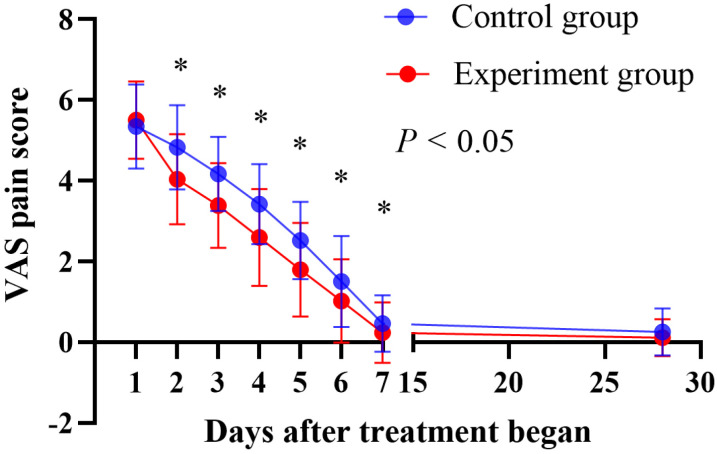
The VAS pain scores of the control and experimental groups on days 1–7 and 28 after treatment began. Comparisons were performed using Pearson’s chi-square or test.

Table 2 presents the VAS pain scores of the control and experimental groups. No difference was observed in the baseline pain scores between the two groups. However, the average pain VAS scores of the experimental group were significantly lower than control group on days 2–7 after treatment began. Interestingly, no significant difference was observed on day 28 after treatment began (Fig 2).

**Table 2:** VAS pain scores between the two groups.

Days after treatment began	Total, median (IQR)	Control Group, median (IQR)	Experimental Group, median (IQR)	*p*-value
1 2 3 4 5 6 7 28	5.00(1.00) 4.00(1.00) 4.00(1.25) 3.00(2.00) 2.00(1.00) 1.00(2.00) 0(0) 0(0)	5.00(1.00) 5.00(2.00) 4.00(1.00) 3.00(1.00) 3.00(1.00v 2.00(2.00) 0(1.00) 0(0)	5.50(1.00) 4.00(2.00) 3.00(1.00) 2.00(1.00) 2.00(1.25) 1.00(2.00) 0(0) 0(0)	0.434 <0.001 <0.001 <0.001 <0.001 0.021 0.013 0.070

VAS: Visual Simulation Scale; IQR: interquartile range

*The VAS pain scores was compared using the Mann-Whitney U test.

### Clinical cure rate between the two groups

**Fig 3:**
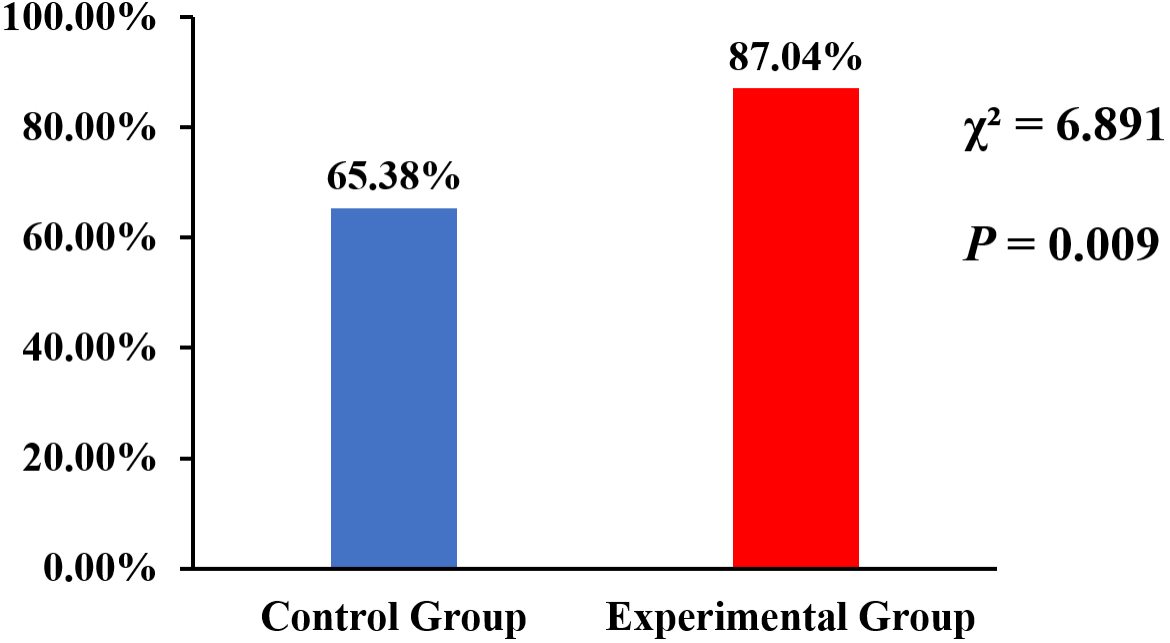
The clinical cure rates of pain for control and experimental groups on days 7 after treatment began.

The clinical cure rates of pain for both groups on days 7 after treatment began are depicted in (Fig 3). The clinical cure rate of pain in the experimental group on days 7 after treatment began showed a 21.66% improvement compared to the control group (87.04% vs 65.38%, p < 0.005).

### DLQI between the two groups

The changes in DLQI score for both groups from pre-treatment to hospital discharge are outlined in Table 3. The DLQI scores did not exhibit significant differences in the pre-treatment phase between the two groups. However, there was significant difference at hospital discharge. Additionally, the DLQI score for the experimental group decreased significantly from pre-treatment to hospital discharge compared to the control group, indicating a statistically significant difference.

**Table 3:** The DLQI scores change for two groups.

Time	Total, median (IQR)	Control Group, median (IQR)	Experimental Group, median (IQR)	*p*-value
Pre-treatment Hospital discharge Change of DLQI	15.50(2.25) 10.00(2.00) 5.00(2.00)	15.00(2.00) 10.00(3.00) 5.00(1.00)	16.00(3.00) 10.00(1.25) 6.00(1.25)	0.586 0.005 <0.001

DLQI: Dermatology Life Quality Index; IQR: interquartile range

*The DLQI score was compared using the Mann-Whitney U test.

### Safety

Table 4 summarizes the side effects observed in both groups. There was no significant difference between the two groups.

**Table 4:** Side effects of two groups.

Side effects	Total (%)	Control Group (%)	Experimental Group (%)	*p*-value
Nephrotoxicity (Level I)	12/106(11.32)	5/52(9.62)	7/54(12.96)	0.627
Hepato-toxicity (Level I)	8/106(7.54)	3/52(5.77)	5/54(9.26)	0.528
Gastrointestinal disorder	21/106(19.81)	12/52(23.08)	9/54(16.67)	0.498
Nausea (Level I)	16/106(15.09)	9/52(17.31)	7/54(12.96)	0.592
Vomit (Level I)	5/106(4.72)	3/52(5.77)	2/54(3.70)	0.632
Upper gastrointestinal bleeding	0	0	0	
Fainting	0	0	0	
Needle break	0	0	0	
Hematoma	0	0	0	

Level I: Define by Common Terminology Criteria for Adverse Events (CTCAE) version 5.0 criteria[29].

* The toxicities was compared using the Pearson’s chi-square test.

## Discussion

WAA was developed by Professor Zhang Xinshu in the 1970s. Compared with conventional acupuncture, WAA offers a simpler approach, only requiring precise needle positioning tailored to the signs and symptoms of the patients. Notably, the needle position in WAA is located in the wrist and ankle, areas devoid of major organs and blood vessels. Despite the location of the needle, WAA demonstrates efficacy in treating a range of systemic problems, particularly pain. WAA is a relatively safe, convenient, and fast method compared to conventional acupuncture [27].

Clinical evidence supports the efficacy of WAA as a stand-alone or in combination with other methods for pain management across different diseases, with minimal side effects. Recent randomized controlled trials have shown that compared to herbal medication alone, WAA combined with herbal medication significantly reduces postpartum abdominal pain, and its mechanism may be related to the up-regulation of serum β-EP level and the increase of pain threshold, leading to analgesia [30]. Additionally, WAA combined with pain nursing care (included pain cognitive intervention, sports and life guidance, posture intervention, psychological intervention) effectively alleviated acute pain associated with urinary calculi and reduced the recurrence rate [22]. The combination of WAA treatment with general anesthesia propofol for painless gastroscopy and colonoscopy reduces propofol dosage, shortens patient recovery time, and reduces related side effects [31, 32]. The role and safety of WAA in reducing acute pain have been confirmed after thyroidectomy, orthopedic surgery, and prostatectomy [24, 25, 33].

This study demonstrated that the experimental group exhibited superior clinical efficacy compared to the control group during the acute phase of HZ without increased side effects. The VAS pain score in the experimental group was lower than that of the control group from days 2–6 after the treatment began. Under consistent baseline VAS pain scores, the addition of WWA significantly improved pain relief in the experimental group compared to the control group. On the 7th day after treatment began, the pain score of the experimental group was lower than that of the control group, but there was no statistical significance, mainly because the scores of both groups were relatively low. However, the pain cure rate of the experimental group on the 7th day after treatment began was significantly higher than that of the control group, and there was statistical significance, it was actually effective on day 7 after treatment began. On the 28th day after treatment began, the pain score of the experimental group was lower than that of the control group, there was no statistical significance, and the pain cure rate was higher than that of the control group, near significant statistical difference on day 28 after treatment began (P = 0.055). The main reason for this was that the wrist ankle acupuncture treatment only lasted for one week.

The DLQI is used to evaluate the impact of skin diseases on the QoL of patients. A previous study found that DLQI was significantly lower in the fire acupuncture group compared to the group receiving herbal medicine alone for psoriasis [34]. Similarly, acupuncture combined with pricking and cupping therapy resulted in significantly lower DLQI scores than traditional therapy in patients with chronic spontaneous urticaria [35]. Additionally, acupuncture reduced DLQI scores in conditions such as atopic dermatitis and rosacea [36–38]. Consistent with these findings, our study observed lower DLQI in the experimental group compared to the control group upon hospital discharge. WWA combined with standard pharmacological treatment improved the QoL of patients during the acute phase of HZ. Thus, the second endpoint of this study was achieved.

Previous studies have established the safety of WAA, similar results have been observed in this study [21–25, 29–34]. Only level 1 nephrotoxicity, hepatotoxicity, and gastrointestinal disorders were observed in this study. Importantly, these effects typically alleviate on their own without needing medical intervention. The addition of WAA did not increase the incidence of side effects.

Our study had certain limitations. First, because wrist ankle acupuncture is an invasive procedure, it was not a double-masked placebo-controlled trial and did not give non-treatment interaction to patients of control group, resulting in the experimental group receive a benefit of additional interaction with healthcare personnel that the control group did not.This may lead to the occurrence of placebo effect, in part because the outcome measures are subjective. Second, While establishing inclusion criteria (VAS ≥ 2), we failed to sufficiently account for the typically severe pain intensity in herpes zoster. Notably, the baseline VAS scores in both the experimental (5.50 (1.00)) and control groups (5.00 (1.00)) align with clinical observations of acute zoster-related pain severity. Third, the underestimated cure rate in the control group led to a smaller-than-ideal sample size. With the observed cure rate and actual sample size, the post-hoc power was 0.78, marginally below the pre-specified threshold of 0.8. However, the chi-square test effect size (Phi coefficient = 0.255) revealed an approximately moderate association between treatment type and outcomes, underscoring the clinical relevance of our findings. Thus, the results still support the conclusion that WAA combined with standard pharmacological treatment provides better pain control. In addition, it was conducted at only one hospital. Fourth, we employed complete randomization to independently allocate participants to the experimental and control groups with a 1:1 allocation ratio. A natural random variation during the randomization process resulted in a final allocation of 54 participants to the experimental group and 52 to the control group. This minor discrepancy is consistent with expected random fluctuations for a total sample size of 106 (under a binomial distribution with p = 0.5, the probability of a ≤ 2-person difference exceeds 80%). All baseline covariates were rigorously compared between groups using chi-square tests (for categorical variables) and Mann-Whitney U test (for continuous variables), confirming no statistically significant imbalances (all p > 0.1).

## Conclusions

To the best of our knowledge, this is the first randomized trial to compare the effects of WAA plus standard pharmacological treatment versus standard pharmacological treatment alone in relieving pain in the acute phase of HZ. The findings suggest that WAA combined with standard pharmacological treatment may potentially improve the pain cure rate at 7 days post-treatment, thus providing a novel approach for pain management in patients in the acute phase of HZ.

## Supporting information

S1 Data CONSORT-2010-Checklist. (DOC)

S2 Data Study protocol Chinese. (DOCX)

S3 Data Study protocol English. (DOCX)

S4 Data Raw Data. (XLSX)
