# Supplementary material for: Wrist-ankle acupuncture alleviates pain in the acute phase of herpes zoster: A randomized controlled trial
Source: PLoS One. 2025 May 29;20(5):e0318386. doi: 10.1371/journal.pone.0318386 (PMC12121775; doi:10.1371/journal.pone.0318386)
Supplement: S2 Data — (DOCX) [file pone.0318386.s002.docx]

（干预性临床研究）

腕踝针减轻带状疱疹急性期疼痛的随机对照研究方案

项目负责人：蒲静

承担科室：绵阳市中心医院中西医结合科

联系电话：13881100019

组长单位：绵阳市中心医院

参加单位：无

研究年限：2023年3月－ 2025年3月

**方 案 摘 要**

| **项目名称** | 腕踝针减轻带状疱疹急性期疼痛的随机对照研究 |
| --- | --- |
| **研究设计**  **(可多选)** | □病例对照研究 □队列研究 □横断面研究  √随机对照研究 □应用盲法 □其他： |
| **研究类型**  **（请根据项目类型勾选）** | **（高风险A）**  □ 基因编辑研究  □ 细胞治疗研究（如干细胞移植）  □ 生物制剂研究  □ 植入性医疗器械研究（含3D打印）  □ 其他（研究者判定，请说明： ）  **（高风险B）**  □ 超药物说明书研究（□超适应症 □超给药途径 □超剂量 □超年龄  □超禁忌症 □超人群 □其他，请说明： ）  □ 超器械说明书研究（□超适应症 □使用范围 □超禁忌症 □超人群  □其他，请说明： ）  □ 其他（研究者判定，请说明： ）  **（高风险C）**  □ 上市后罕见病药物研究（孤儿药）  □ 上市后治疗性疫苗研究  □ 已上市药物研究（包括化药、仿制药等）  □ 已上市器械研究（含AI，影像软件）  □ 新组合治疗研究  □ 新术式研究  □ 特殊人群研究（儿童、孕妇、智力低下者、精神障碍受试者等）  □ 其他（研究者判定，请说明： ）  **（低风险）**  □ 既往临床数据回顾性研究  □ 既往临床标本回顾性研究  □ 建立标本库研究  □ 个案病例报道  □ 非植入性医疗器械研究（如面罩、牙垫等）  √其他（研究者判定，请说明：未纳入禁止类技术和限制类技术目录的医疗新技术运用 ） |
| **研究方法**  **(可多选)** | □文献研究法 □实地调查法 □问卷调查法 □信息分析法 √对比分析法 √数据分析法 □统计分析法 □案例分析法 |
| **病例总数** | 106人 |
| **风险判断** | √不大于最低风险 □大于最低风险 |
| **研究期限** | 2023年3月 20 日 至 2025 年 3 月 20 日 |

**一、研究背景**

带状疱疹(Herpes zoster，Hz)是由水痘-带状疱疹病毒(Varicella-zostervirus，VZN)所引起，一般民间又称为“蛇串疮”，其全球患病率为0.14％-0.48％，病因为既往感染的病毒潜伏在脊髓后根神经节的神经元中，当病毒再次激活时导致皮肤、神经发生损坏。

流行学研究提示随着带状疱疹的发病率日益增多，带状疱疹后遗神经痛的患者也在增多，这引起了广泛关注。不同国家的HZ流行特征均显示50岁及以上人群发病率上升明显，≥50岁的人群，免疫功能低下和慢性病人群都是带状疱疹HZ的高发人群^[1]^。正常状态下仍有4％的复发率，若遇机体免疫功能低下状态，可以高达8％的复发率，近年来由于各种生活环境，社会因素导致的身心压力增加及糖皮质激素与免疫调节类药物使用量的提高，使得本病的发生率有逐年向上的趋势^[2-3]^。

此疾病由病毒所引起，受损伤的部位为神经和其节段相关的皮肤，及相邻组织，故西医在治疗带状疱疹以抗病毒，营养神经以及缓解疼痛为主要治疗手段^[4-6]^。其中缓解疼痛常用药物主要有，甾体类抗炎药、抗焦虑抑郁药物、抗癫痫药物、和轻中度阿片药物等。其它如糖皮质激素、神经阻滞术及各种物理疗法^[7]^，对带状疱疹急性期疼痛也有一定的疗效。当皮损脱痂后，疼痛超过4周遗留后遗神经痛，服用非甾体类、抗癫痫药，甚至阿片类药物，虽然能有所缓解，但是长期服用副作用大，比如损伤胃黏膜导致胃出血、呼吸抑制、心率失常、肝肾损害等，给病人带来极大的痛苦^[8]^。

传统医学将蛇串疮大致区分成：肝经火盛、肝郁气滞血瘀以及脾经湿热等类型^[9]^。但不论分型如何，总以疼痛为病患最急切想解决的问题，所以如果能够解决带状疱疹带来的疼痛，带状疱疹患者基本上是可以正常作息，并不会影响生活质量。在缓解疼痛方面，针灸一直有良好的效果而被世人推崇，针灸在研究带状疱疹疼痛的控制方面，通常以电针、围刺、火针、铺棉灸、刺络拔罐等治疗方法加以混合治疗，各研究均取得不错的疗效^[10-14]^。然而各种疗法相加耗时60-70分钟，且影响患者活动，在临床上不易推广。因此寻找一种镇痛效果好，起效快，不良反应少，患者乐于接受的镇痛方法刻不容缓。

据文献报道，腕踝针(Wist-ankleacupuncture)可通过皮下浅刺法，对于疼痛类疾病有较好的止痛作用^[15]^。腕踝针疗法是近几十年来极具特色的新兴疗法，指在特定的腕踝部分下选取进针点把毫针循肢体纵轴沿真皮下刺入到一定的长度以治疗疾病。

腕踝针针刺技术与传统毫针针刺手法相比：其针刺为沿身体纵轴进行皮下浅刺，其刺激深度位于皮下，归属十二皮部范畴，且不要求患者出现酸、麻、重、胀等感觉，这种痛觉极其微小的针刺方法很好地规避了垂直针刺的深度不易把控、针感过于强烈以及滞针等问题。且腕踝针技术针刺部位位于手腕部及脚踝部，避开了重要脏器、大血管、筋脉循序处，行针、留针时安全可靠^［16］^。

基于针刺留针研究表明，运用针灸治疗时其留针时间持续20分钟以上止痛效果才能充分发挥。又有最新研究指出，较长的留针时间治疗痛症临床疗效优于留针时间较短者。如许云祥^[17]^等分别留针5分钟、20分钟、30分钟、40分钟和60分钟来治疗急慢性踝关节软组织损伤，结果显示留针30分钟对急性最佳，留针60分钟对慢性为最优。

腕踝针作为治疗痛症的一种特殊针刺方法^[18]^，有用于治疗带状疱疹疼痛的报道，但是病例数较小，且无严格的随机对照研究来证实^[19,20]^。本研究拟采用严格的随机对照方法，来确定在基础治疗（抗病毒治疗+非甾体药物止痛）的基础上加腕踝针能否提高带状疱疹急性疼痛治疗的显效率，期望为带状疱疹急性疼痛提供一种新的治疗策略。

**二、研究目的**

1. 主要目的：通过临床随机对照试验，观察腕踝针缓解带状疱疹急性期疼痛的临床疗效。

2. 次要目的：无

**三、研究设计类型与研究步骤**

1. 研究设计

1.1研究方法：本研究将采用前瞻性随机对照试验

1.2研究对象

本研究按照纳入、排除标准，选取我院皮肤科带状疱疹急性期的住院患者为研究对象。

1.3样本量的估算

对于随机对照试验样本量的计算采用的是pass软件中两个独立样本随机对照试验。权重β取80%;α取0.05：

（1）检索文献里已知治疗的有效率设定为P2，查阅文献得知，抗病毒治疗+非甾体药物止痛P2为60%。

（2）根据既往文献，我们预计治疗组能够达到的有效率P1为85%。得到随机对照的每组需要48名患者，再按照10%脱失率,每组需要53名病人。

1.4分组方法

根据纳入排除标准，将符合标准的106例患者，采用随机数字表的方法，通过电脑分配随机数字表，对取得的随机数字简单随机分组，分为腕踝针组(53例)与对照组(53例)，研究者根据电脑分配随机数字表的分组情况给于相应方案的治疗。

1.5诊断标准

参照中华医学会《临床诊疗指南·皮肤病与性病分册》2006年版带状疱疹的诊断标准。

①发疹前可有疲倦、低热、全身不适、食欲不振等前驱症状：

②患处有神经痛，皮肤感觉过敏：

③好发部位是肋间神经、三叉神经、臂丛神经及坐骨神经支配区域：

④皮疹为红斑上簇集性粟粒至绿豆大水疱，疱液常澄清：

⑤皮疹常单侧分布，一般不超过躯体中线：

⑥病程有自限性，约2-3周，愈后可遗留色素改变，发生坏死遗疡者可以留瘢痕：

⑦头面部带状疱疹可累及眼耳部，引起疱疹性角膜结膜炎或面瘫等。

2. 研究步骤


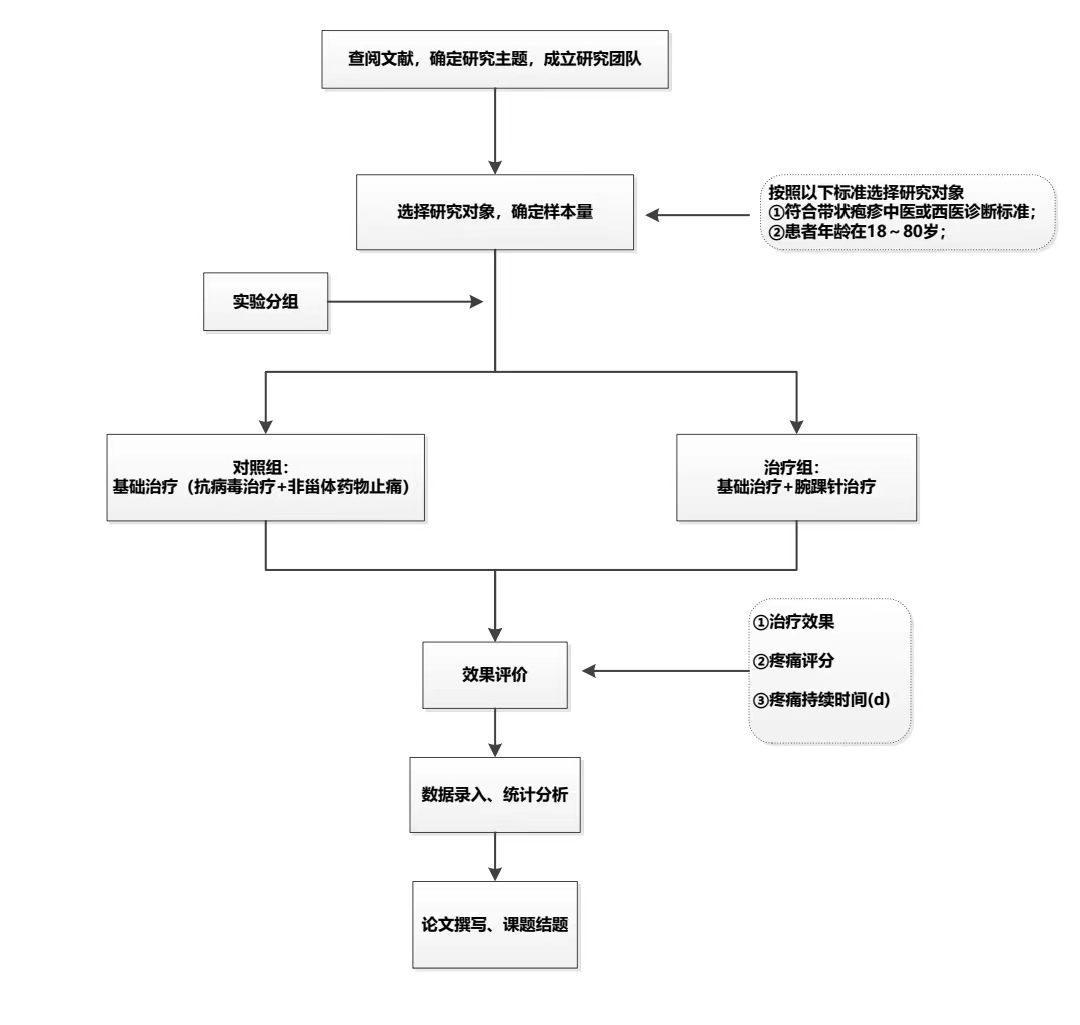


**四、病例选择**

1. 入选标准

①符合带状疱疹诊断标准的住院病人；②皮疹具有不同程度的疼痛，VAS评定法大于等于2分；③患者年龄在18～80岁；④病程在1-7天，且未经过抗病毒、糖皮质激素等药物治疗；⑤接受本课题组治疗方法并签署知情同意书者。符合以上所有条件者方可纳入。

2. 排除标准

①特殊类型的带状疱疹，如眼、耳带状疱疹、内脏带状疱疹、带状疱疹性脑膜炎、泛发性带状疱疹、无疹型带状疱疹；②有心脑血管疾病、肝肾损害、血小板减少、凝血功能异常、恶性肿瘤等基础病；③妊娠或哺乳期妇女。凡符合以上任何一项者均应排除。

3. 剔除标准

试验过程中发现符合排除标准者或不符合纳入标准，或因其他因素，导致患者不能继续按照本次研究方案进行治疗。

4.中止研究标准

如在研究过程中发现临床研究方案制定有重大失误，观察指标不能客观的评价治疗效应，或者实施方案的过程中发现缺乏科学性，难以评价疗效：或者是研究中患者反馈总体治疗效果不佳，甚至病情加重，不具有临床价值者。

1. **研究方法**
2. 研究（干预）方案

对照组（标准治疗：抗病毒治疗+止痛药物)、治疗组（标准治疗+腕踝针区域留针30min)。治疗组腕踝针疗程为每组患者每天分别干预1次，持续干预7天(1个疗程)

腕踝针干预方法

①操作者为专职中医专科护士，熟练掌握腕踝针操作手法。

②患者与施针者体位

针刺时协助患者取仰卧位、侧卧位，一般在临床上经常取仰卧位。患肢要和正前方相对，面向扎针者，患肢肌肉尽可能保持放松状态。扎针者应该和患者被针刺肢体保持在同一水平线上，便于观察针刺入皮下时是否处于同一水平线上。

③进针点的位置与针刺方向

根据带状疱疹疼痛部位选择区域，再根据针刺点的选区方法来确定进针点，身体分区：以前后中线为界，身体两侧由前向后各分6个纵区，用数字1~6编号，用于症状定位。如图2


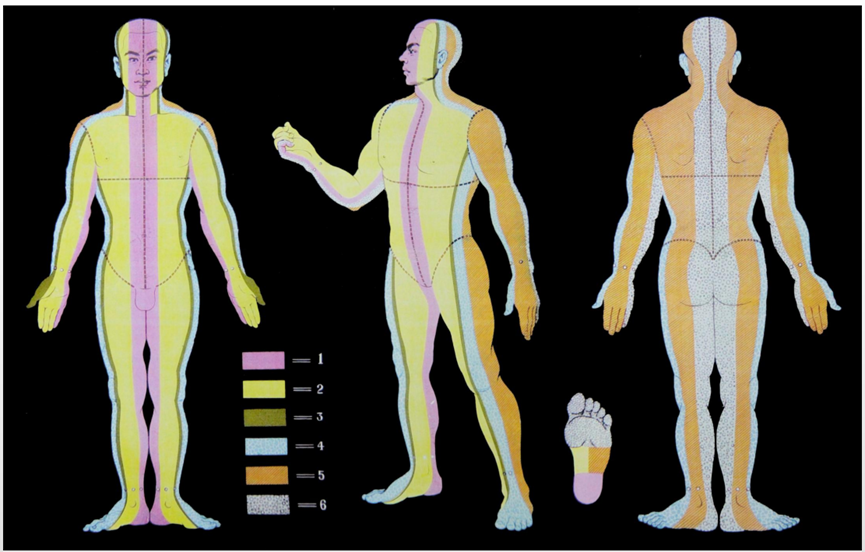


图2

手腕部及脚踝部的进针部位：

手腕部：排列在腕横纹以上约二横指环腕一圈处，记作上1~6。如图3


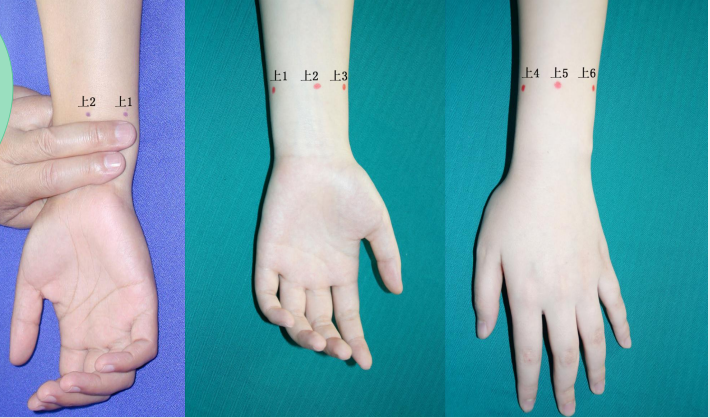


图3

脚踝部：排列在内踝和外踝上约三横指环踝一圈处，记作下1~6。如图4


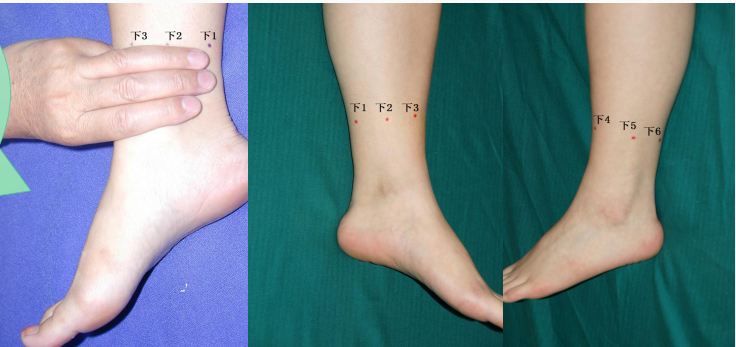


图4

在扎针的过程中，要根据皮肤的实际情况适当调整进针点的位置，比如针刺局部皮肤是否有瘢痕，针刺的方向等，使针刺入皮下达到适当的长度，即进针点并不是绝对固定的。

④腕踝针的操作流程

在干预之前，避开针刺点局部血管，确定选区后，以进针点为中心，用75％酒精常规消毒进针点周围皮肤2遍后，腕踝针以30度刺入皮下，确认针尖刺人真皮层后，轻捻针柄，使针沿着皮下表浅位置缓慢进针，直至针体露出针身2mm时停止进针，然后用胶带妥善固定针柄。进针时以感觉松而没有阻力为宜，并且患者没有任何酸、麻、胀、痛等特殊感觉为宜：如果在穿刺过程中引起酸、麻、胀、痛，要根据患者的主诉调整进针的角度，重新进针并固定好。

1. 研究用药/器械名称、规格（如涉及）

采用蜀康牌0.25mmx25mm毫针，即为1寸的毫针，生产厂家为长春爱康医疗器械有限公司。

1. 合并用药物

可合并使用药物：抗病毒药物、非甾体止痛药物

1. **检测项目与检测时点**

不涉及

1. **疗效评定标准**

1、治疗效果：参照1993年卫生部颁布的“中药新药临床研究指导原则” 执行。临床治愈：疼痛消失；显效：疼痛强度减轻2分；有效：疼痛强度减轻1分；无效：疼痛强度减轻不足1分。显效率=显效数 /总例数× 100%。

2、疼痛评分：根据中华医学会疼痛学会监制的疼痛视觉模拟尺评法(VAS评定法，单位：mm)记录观察点前24小时内最痛点。0分：无痛：3分以下：有轻微的疼痛，能忍受：4分-6分：患者疼痛并影响睡眠，尚能忍受；7分-10分：患者有渐强烈的疼痛，疼痛难忍，影响食欲，影响睡眠。

3、疼痛持续时间(d)：从患者开始感觉有疼痛到疼痛完全消失所需时间。

两组均于试验第1、3、5、7天每次治疗前和疼痛的第28天（以开始出现疼痛为第1天计算)记录①、②、③项，如③项在疼痛的第28天（疼痛未缓解或者消失)未能记录，则记录为28天。两组疼痛缓解强度和显效率以治疗后疼痛缓解最好分数计算。

1. **不良事件的观察及处理**

不良事件定义为由医疗导致的伤害，与疾病的自然转归相反，延长了病人的住院时间，导致残疾的一切事件，包括可预防和不可预防的不良事件。不可预防的不良事件指正确的医疗行为造成的不可预防的损伤；可预防的不良事件指医疗中由于未能防范的差错或设备故障造成的损伤。

1、皮下出血

腕和踝是活动较多的部位，又处于四肢末端，动静脉交错，血液供应丰富，皮下静脉网多，血管分布因人而异，皮下脂肪层薄者较粗静脉血管尚能看清，针刺时可避免，但脂肪层较厚者皮下血管多不易辨认，针刺难免伤及血管，而出现皮下出血。

处理方法：

为了预防皮下出血，进针时尽量避免伤及可见较粗的静脉，进针要缓慢。如果在进针过程中患者感觉进针处疼痛，可能为针尖触及血管壁，必须将针略微退出一点，尝试更表浅地刺入；若发现针尖部皮肤缓慢隆起，表示已有出血，要立即拔出针灸针，并压迫止血如已有皮下出血，应向患者说明以消除其顾虑。

2、可能会出现晕针，从而导致跌倒、休克等现象。

处理方法：

晕针发生时，患者先感觉恶心乏力，头昏，或有耳鸣，视力模糊，或感觉眼前发黑，面色变苍白，出冷汗。继之呼吸表浅，口唇发绀，意识不清，不能站立，倒地，呈休克状态。

一旦出现晕针现象，必须立即停止针刺，拔出针灸针，让患者立即平卧；解开患者衣领，保持呼吸通畅：注意血压变化：或给患者喝温开水或糖水，必要时给予吸氧，一般数分钟之内可以恢复正常。

**九、研究的质量控制与质量保证**

影响研究因素主要有：选择性偏倚、衡量偏倚、混杂偏倚，由于该试验设计的特殊性，无法做到盲受试者，为非盲法状态下观察，极易发生衡量偏倚。为控制好研究质量，减少偏倚，应严格遵照随机对照方案进行，严格限制研究对象，严格执行纳入、排除标准，试验过程中严格控制质量。

1、使用同一批次的毫针

在干预的过程中用统一购买的采用蜀康牌0.25mmx25mm毫针，即为1寸的毫针，生产厂家为长春爱康医疗器械有限公司。

2、研究人员分工及相关人员培训

课题组成员由培训人员、评估人员、干预人员组成。培训人员负责培训腕踝针的相关知识及穿刺手法，对评估单的内容及干预流程进行审核。评估人员由1名护理研究生，2名主管护师组成，对带状疱疹患者进行评估，并发放、收集资料。干预人员由中医专科护士对纳入的研究对象进行干预，在干预的过程中，严格按照标准的操作流程进行操作。

干预前应对患者进行腕踝针相关知识的培训，特别对疼痛相关知识进行培训，告知疼痛程度的真实反映和正确表达疼痛程度的方法等。

3、在试验过程中严格控制质量：严格随机化分组：对纳入的研究对象，根据随机数字表进行随机分组；资料由三名经过培训的课题组成员统一进行收集控制试验偏倚；数据核对后双人录入。

**十、数据安全监查**

临床研究将根据风险大小制定相应的数据安全监察计划。所有不良事件均详细记录，恰当处理并追踪直到妥善解决或病情稳定，按照规定及时向伦理委员会、主管部门、申办者和药品监督管理部门报告严重不良事件与非预期事件等；主要研究者定期对所有不良事件进行累积性回顾，必要时召开研究者会议评估研究的风险与受益；双盲试验必要时可以进行紧急揭盲，以确保受试者安全与权益。大于最小风险的研究将安排独立的数据监察员对研究数据进行监查，高风险研究将建立独立的数据安全监察委员会对累积的安全性数据以及有效性数据进行监查，以做出研究是否继续进行的建议。

**十一、统计学处理**

采用SPSS22.0进行统计分析，符合正态分布的计量资料用均数±标准差进行描述，不符合正态分布采用M(P25～P5)进行统计描述，符合正态分布且方差齐的计量资料采用F检验分析四组间的差异，符合正态分布但方差不齐采用秩和检验：不符合正态分布的，采用Kruskal-Wallis秩和检验分析四组间差异，及两两比较差异：治疗前后组内差异并且符合正态分布采用配对设计两独立样本·检验，不符合正态分布采用两独立样本秩和检验，P＜0.05为差异有统计学意义。

**十二、临床研究伦理原则与要求**

临床研究将遵循世界医学大会《赫尔辛基宣言》、国家卫健委《涉及人的生物医学研究伦理审查办法》《医疗卫生机构开展研究者发起的临床研究管理办法》等相关规定。在研究开始之前，由伦理委员会批准该试验方案，且临床研究的有关信息提前30日在医学研究登记备案信息系统备案后方可正式启动。

每一位受试者入选本研究前，研究者有责任向受试者或/和其法定监护人完整、全面地介绍本研究的目的、程序和可能的风险，并签署书面知情同意书，应让受试者知道他们参加本项研究是完全自愿的，他们可以拒绝参加研究，也可以参与后在试验任何阶段随时退出，他们不会受到不公正的待遇，不会影响与临床医师/护士的关系，也不会影响他们的正常治疗。知情同意书应作为临床研究文件保留备查。研究过程中将保护受试者的个人隐私与数据机密性。

**十三、研究进度**

| 开始时间 | 结束时间 | 阶段目标 |
| --- | --- | --- |
| 2023-01-01 | 2024-6-30 | 开始实验入组病人，治疗和收集相关临床资料，完成所有病例入组。 |
| 2024-07-01 | 2024-9-30 | 对前一年度收集的资料进行总结，运用统计软件进行分析。 |
| 2024-10-01 | 2024-12-31 | 总结研究成果，完成结题。 |

**十四、参加人员**

| **姓名** | **职称** | **专业** | **任务** | **GCP培训（时间）** |
| --- | --- | --- | --- | --- |
| 杜小波 | 主任医师 | 肿瘤 | 课题设计、监督、论文修改 | 有（2020） |
| 李代文 | 主管护师 | 中医护理 | 课题设计、统计、论文撰写 | 无 |
| 邓兰兰 | 主管护师 | 护理管理 | 技术指导 | 无 |
| 罗霞 | 主管护师 | 护理管理 | 统计,资料收集及录入、论文撰写 | 无 |
| 谭冬梅 | 主治医师 | 中医皮肤 | 项目实施与资料收集 | 无 |
| 何沅莉 | 主治医师 | 皮肤临床 | 项目实施与资料收集 | 无 |
| 王娟 | 主管护师 | 中医护理 | 项目实施与资料收集、论文撰写 | 无 |
| 李燕霞 | 主管护师 | 护理学 | 项目实施与资料收集、论文撰写 | 无 |
| 赵贤坤 | 医师 | 中医临床 | 项目实施与资料收集、论文撰写 | 无 |

**十五、主要参考文献**

[1]李娟,吴疆.带状疱疹的流行病学和疫苗免疫策略[J].慢性病学杂志,2021,22(08):1145-1151.DOI:10.

16440/J.CNKI.1674-8166.2021.08.01.

[2] Marra F, Parhar K, Huang B, et al. Risk factors for herpes zoster infection: a meta- analysis [J]. Open Forum Infect Dis, 2020, 7(1):ofaa005.

[3] Torcel- Pagnon L, Bricout H, Bertrand I, et al. Impact of underlying conditions on zoster- related pain and onquality of life following zoster [J]. J Gerontol A Biol Sci Med Sci, 2017, 72(8):1091-1097

[4]魏敏,闫言.带状疱疹的药物治疗进展[J].临床药物治疗杂志,2019,17(11):33-37.

[5]王燕清,孟祥慧.糖皮质激素治疗带状疱疹的应用进展[J].牡丹江医学院学报,2021,42(02):141-143.DOI:10.13799/j.cnki.mdjyxyxb.2021.02.037.

[6]吉宁,赵行,曾昕,陈谦明.核苷类抗疱疹病毒药物的研究进展[J].国际口腔医学杂志,2018,45(03):351-357.

[7]易维君,王珍珍,张林,李莉.半导体激光治疗带状疱疹后遗神经痛研究进展[J].激光杂志,2020,41(11):203-205.DOI:10.14016/j.cnki.jgzz.2020.11.203.

[8]段苡文,郭书萍.带状疱疹后遗神经痛研究进展[J].中华老年多器官疾病杂志,2019,18(07):552-556.

[9]范瑞强.带状疱疹[M].北京：中国中医药出版社2012：16-17.

[10]李茜,吴明霞.中医外治法治疗带状疱疹治法综述[J].中国医药导刊,2020,22(12):865-869..

[11]庄婷,刘雪梅,王亚萍,龙雅静,唐荣,李思梦,王伟.黄帝内针联合阿昔洛韦治疗头面部带状疱疹急性期的临床研究[J/OL].中医临床研究:1-3[2022-09-18].https://er.szlib.org.cn:443/rwt/331/http/NNYHGLUDN3WXTLUPMW4A

/kcms/detail/11.5895.R.20220815.1013.006.html

[12]王娜娜,王伟,王海泉.中医外治法治疗带状疱疹后遗神经痛研究概况[J].实用中医药杂志,2021,37(01):154-155.

[13]陈庆. 刺络放血疗法治疗带状疱疹相关性疼痛的临床观察[D].湖北中医药大学,2022.DOI:

10.27134/d.cnki.ghbzc.2022.000374.

[14]黄高振,王亮,吴婧瑄,吴博,邓杰,黄筱钧.传统中药、针法与西药抗病毒治疗带状疱疹的疗效比较[J].湖北民族大学学报(医学版),2021,38(04):16-20.DOI:10.13501/j.cnki.42-1590/r.2021.04.004.

[15]杨克,杜玉茱,石晶,王建岭,孙彦辉,邢海娇,李晓峰,徐晶,张选平,张莘,贾春生.利用数据挖掘技术探析腕踝针疗法的优势病种及临床应用特点[J].中国针灸,2019,39(06):673-678.DOI:10.13703/j.0255-2930.2019.06.029

[16]兰蕾，张国山．腕踝针疗法［M］．2 版．北京:中国医药科技出版社，2012．

[17]许云祥,陈贵珍.不同留针时间对踝关节软组织损伤的疗效观察[J].中国针灸,2001(10):33-34.

[18]郭珍妮,陈致尧,许精鑫,彭德忠.近5年腕踝针临床应用现状及其规律分析[J].按摩与康复医学,2020,11(08):56-59.DOI:10.19787/j.issn.1008-1879.2020.08.021.

[19] 毛光兰，齐国豪，张新颖，等． 不同留针时间针刺治疗带状疱疹后遗神经痛疗效观察［J］． 新乡医学院学报，2012，29( 7) : 514-516．

[20] 樊玲，张灵，曾令川. 超时留针和刺络加强治疗带状疱疹后遗神经痛的临床观察［J］．四　川　中　医，2006，24（5），107-108.
