# Supplementary material for: Wrist-ankle acupuncture alleviates pain in the acute phase of herpes zoster: A randomized controlled trial
Source: PLoS One. 2025 May 29;20(5):e0318386. doi: 10.1371/journal.pone.0318386 (PMC12121775; doi:10.1371/journal.pone.0318386)
Supplement: S3 Data — (DOCX) [file pone.0318386.s003.docx]

(Interventional clinical study)

Wrist-ankle acupuncture alleviates pain in the acute phase of herpes zoster: a randomized controlled trial

Project Leader: Pu Jing

Department in Charge: Integrated Traditional Chinese and Western Medicine Department of Mianyang Central Hospital

Contact Number: 13881100019

Leading Unit: Mianyang Central Hospital

Participating Units: None

Duration of Study: March 2023 - March 2025

**Protocol Summary**

| **project name** | Wrist-ankle acupuncture alleviates pain in the acute phase of herpes zoster: a randomized controlled trial |
| --- | --- |
| **research design**  **(Can be selected more)** | □ Case-control study □ cohort study □ cross-sectional study  √Randomized controlled study □ Application of blinding Other: |
| **The type of research**  **(Check according to project type)** | **(highrisk A)**  □ Gene-editing studies  □ Cell therapy study (e. g., stem cell transplantation)  □ Biologics study  □ implantable medical device study (including 3D printing)  □ Others (for the investigators judgment, please specify:)  **(highrisk B)**  □ Off-label study (□ off-indication □ overdose route □ off-dose □ over age  □ Supernormal contraindications □ superpopulation □ Other, please specify:)  □ Off-device specification study (□off-indication □rangeofuse □super-contraindication □ off-population  □ Others, please specify:)  □ Others (for the investigators judgment, please specify:)  **(highrisk C)**  □ Post-market drug study for rare diseases (orphan drug)  □ Post-marketing therapeutic vaccine study  □Listed drug studies (including chemical drugs, generic drugs, etc.)  □Listed device study (including AI, imaging software)  □ New combination therapy study  □New surgical research  □ Special population studies (children, pregnant women, people with low intelligence, subjects with mental disorders, etc.)  □ Others (for the investigators judgment, please specify:)  **(low risk)**  □Previous clinical data were retrospective study  □Retrospective study of previous clinical specimens  □ Establish a specimen library study  □ Case cases reported  □ Study of non-implantable medical devices (such as face mask, dental pad, etc.)  √Others (according to the investigator, please explain: application of new medical technologies not included in the list of prohibited and restricted technologies) |
| **research technique**  **(Can be selected more)** | □Literature research method □field survey method □questionnaire survey method □information analysis method √comparative analysis method √data analysis method □statistical analysis method □case analysis method |
| **Total number of cases** | 106 People |
| **risk judgement** | √No greater than the minimum risk □ greater than the minimum risk |
| **The study period** | From 20 March 2023 to 20 March 2025 |

**I. Research Background**

Herpes zoster (Hz) is caused by the varicella-zoster virus (VZN), commonly known as "shingles" in folk culture, with a global prevalence rate of 0.14%-0.48%. The cause is the latent virus in the spinal ganglion neurons, which reactivates to cause damage to the skin and nerves.

Epidemiological studies suggest that with the increasing incidence of herpes zoster, the number of patients with postherpetic neuralgia is also increasing, which has attracted widespread attention. The epidemic characteristics of HZ in different countries all show a significant increase in incidence in people over 50 years old, and people with low immune function and chronic diseases are high-risk groups for herpes zoster HZ[1]. Normally, there is a recurrence rate of 4%, which can reach up to 8% in the case of low immune function, and in recent years, due to various living environments and social factors leading to increased mental and physical stress and the increased use of glucocorticoids and immune modulating drugs, the incidence of this disease has been increasing year by year[2-3].

This disease is caused by a virus that affects the nerves and the skin and adjacent tissues related to the nerve segments, so Western medicine mainly treats herpes zoster with antiviral, nerve nutrition, and pain relief as the main treatment methods[4-6]. Common pain-relief drugs include steroidal anti-inflammatory drugs, anti-anxiety and antidepressant drugs, anti-epileptic drugs, and mild to moderate opioid drugs. Other treatments such as glucocorticoids, nerve blockage, and various physical therapies[7] also have certain effects on acute pain of herpes zoster. When the skin lesion scabs off and the pain lasts more than 4 weeks, leaving postherpetic neuralgia, taking non-steroidal, anti-epileptic, and even opioid drugs can provide some relief, but long-term use has significant side effects, such as gastric mucosal damage leading to gastric bleeding, respiratory depression, heart rate disorders, and liver and kidney damage, causing great suffering for patients[8].

Traditional medicine roughly divides snake sores into types such as liver meridian fire excess, liver stagnation and qi stagnation with blood stasis, and spleen meridian damp-heat[9]. Regardless of the type, the most urgent problem for patients is pain, so if the pain caused by herpes zoster can be resolved, patients with herpes zoster can basically have a normal routine and will not affect the quality of life. In terms of relieving pain, acupuncture has always been well received and is highly regarded. Acupuncture in the study of controlling herpes zoster pain is usually treated with a combination of electroacupuncture, encircling needling, moxibustion, cotton padding moxibustion, and bloodletting cupping, and each study has achieved good results[10-14]. However, the combination of various therapies takes 60-70 minutes and affects patient activity, which is not easy to promote in clinical practice. Therefore, it is urgent to find a pain relief method that is effective, fast-acting, has few adverse reactions, and is readily accepted by patients.

According to literature reports, wrist-ankle acupuncture (Wist-ankleacupuncture) can have a good analgesic effect on pain diseases through subcutaneous shallow needling[15]. Wrist-ankle acupuncture is a distinctive new therapy in recent decades, which means selecting acupuncture points in specific wrist and ankle parts and inserting filiform needles along the body's longitudinal axis under the skin to a certain length to treat diseases.

Compared with traditional filiform needle acupuncture techniques, wrist-ankle acupuncture is a subcutaneous shallow needling along the body's longitudinal axis, with the stimulation depth located under the skin, belonging to the twelve skin parts category, and does not require patients to experience sour, numb, heavy, and swollen feelings. This minimally painful acupuncture method well avoids the issues of vertical needling depth being difficult to control, overly strong needle sensation, and needle retention problems. Moreover, the acupuncture parts of wrist-ankle acupuncture are located on the wrist and ankle, avoiding important organs, large blood vessels, and meridian sequences, making the needling and needle retention safe and reliable[16].

Based on acupuncture needle retention studies, the analgesic effect of acupuncture treatment can only be fully exerted when the needle retention time lasts for more than 20 minutes. The latest research points out that a longer needle retention time is clinically more effective in treating pain than a shorter retention time. For example, Xu Yunxiang[17] and others treated acute and chronic ankle joint soft tissue injuries by retaining the needle for 5 minutes, 20 minutes, 30 minutes, 40 minutes, and 60 minutes, respectively. The results showed that a 30-minute needle retention was optimal for acute cases, and a 60-minute needle retention was optimal for chronic cases.

Wrist-ankle acupuncture, as a special acupuncture method for treating pain[18], has been reported to treat herpes zoster pain, but the number of cases is small, and there is no strict randomized controlled study to confirm[19,20]. This study plans to use strict randomized controlled methods to determine whether wrist-ankle acupuncture can improve the curative effect of acute pain treatment of herpes zoster on the basis of basic treatment (antiviral treatment + non-steroidal drug analgesia), hoping to provide a new treatment strategy for acute pain of herpes zoster.

**II. Research Purpose**

1. Main objective: To observe the clinical efficacy of wrist and ankle needle in relieving acute pain of herpes zoster through clinical randomized controlled trial.

2. Secondary purpose: None

**III. Research Design Type and Research Steps**

**1. Study design**

1.1 Study method: This study will be a prospective randomized controlled trial

1. 2 Study subjects

In this study, we selected inpatients in the acute phase of herpes zoster in the dermatology department of our hospital.

1. 3 Sample size estimation

For the sample size calculation of randomized controlled trials, two independent sample randomized controlled trials in the pass software were used. Weight β 80%; α 0.05:

(1) The response rate of known treatment in the search literature was set to P2, and the literature showed that antiviral therapy + nonsteroidal drug analgesic P2 was 60%.

(2) Based on previous literature, we expect the effective rate P1 to be 85%. There were 48 patients per group for the randomized control, and at another 10% loss rate, 53 patients for each group.

1. 4 Group grouping method

According to the inclusion and exclusion criteria, 106 patients meeting the criteria, using the method of random number table, through the computer random number, divided into wrist and ankle needle group (53 cases) and the control group (53 cases), the investigators according to the computer allocation random number table group to the corresponding scheme of treatment.

1. 5 Diagnostic criteria

Refer to the diagnostic criteria of herpes zoster in 2006 edition of Clinical Diagnosis and Treatment Guidelines · Dermatology and Venereal Diseases " of the Chinese Medical Association.

① Before the rash can have fatigue, low fever, general discomfort, loss of appetite and other prodrome symptoms:

② With neuralgia, skin sensory allergy:

③ The best hair site is the intercostal nerve, trigeminal nerve, brachial plexus nerve and sciatic nerve innervation area:

④ The rash is a cluster of millet to mung bean blister, blister fluid is often clarified:

⑤ The rash is often distributed unilaterally, generally not exceeding the somatic midline:

⑥ The course of the disease is self-limited, about 2-3 weeks, after healing can be left pigment change, necrosis can leave scar:

⑦ Head and facial herpes zoster can involve the eyes and ears, causing herpetic keratoconjunctivitis or facial paralysis.

**2. Study steps**

**IV. Case selection**

**1. Inclusion criteria**

① Inpatients meet the diagnostic criteria of herpes zoster in Chinese medicine or Western medicine; ② rash has different degrees of pain, VAS assessment method greater than or equal to 2; ③ patients aged 18 to 80 years; ④ duration for 1-7 days, and without antiviral or glucocorticoids; ⑤ received the treatment of the research group and signed the informed consent. Only those who meet all of the above conditions can be included.

**2. Exclusion criteria**

① Special types of herpes zoster, such as eye, ear herpes zoster, herpes zoster, meningitis, generalized herpes zoster, herpes zoster; ② has cardiovascular and cerebrovascular diseases, liver and kidney damage, thrombocytopenia, abnormal coagulation function, malignant tumors and other basic diseases; ③ pregnant or lactating women. Any compliance with any of the above shall be excluded.

**3. Elimination criteria**

During the trial, those found to meet the exclusion criteria or not to meet the inclusion criteria, or other factors caused the patient to continue treatment according to the study protocol.

**4. Discontinuation of the study criteria**

For example, it is found in the process of the research that there are major mistakes in the formulation of the clinical research plan, the observation indicators can not objectively evaluate the treatment effect, or the implementation of the plan is difficult to evaluate the curative effect, or the overall treatment effect is not good, or even the condition is aggravated, and it does not have clinical value.

**V.research technique**

**1.Study (intervention) protocol**

Control group (standard pharmacological treatment: antiviral therapy combined with pain relievers drugs), treatment group standard pharmacological treatment + wrist and ankle acupuncture for 30min). The course of wrist and ankle injection in the treatment group was conducted once per day in each group for 7 days (1 course)

Wrist and ankle needle intervention method

① The operator is a full-time Chinese medicine nurse, skilled in wrist and ankle needle operation.

② Position of the patient and the user

When acupuncture to assist the patient to take the supine position, lateral position, generally in clinical often take the supine position. The affected limb should be opposite the front of the needle, and the muscles should be relaxed as much as possible. The needle should be kept on the same level as the patient to see whether the needle is on the same level when inserted subcutaneously.

③ The position of the needle injection point and the direction of the needle injection

Select the area according to the pain site of herpes zoster, and then determine the needle entry point according to the selection method of the needle prick point, the body partition: the boundary is the boundary, the two sides of the body are divided into 6 vertical areas from front to back, with the numbers 1 to 6, for symptom positioning. Figure 2


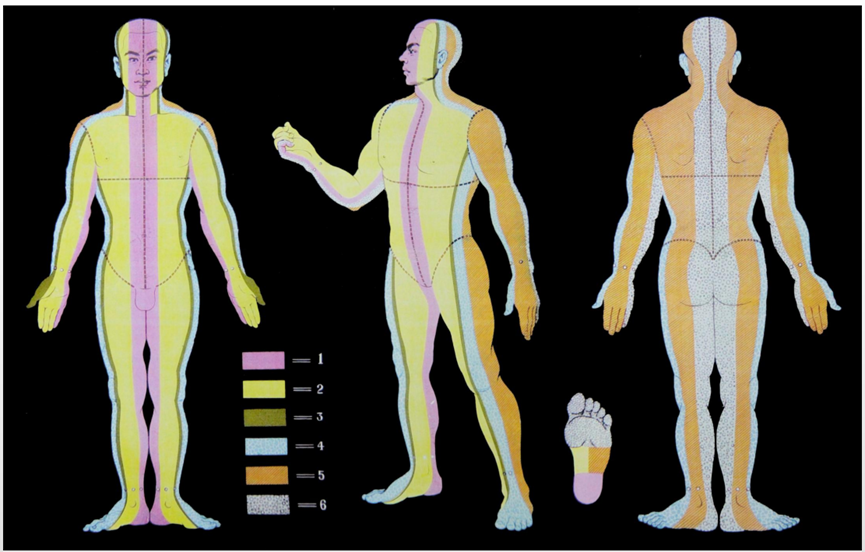


Figure 2

Needlet at wrist and ankle:

Wrist: arranged in the wrist above about two horizontal ring wrist circle, recorded as 1~6. As shown in Figure Figure 3


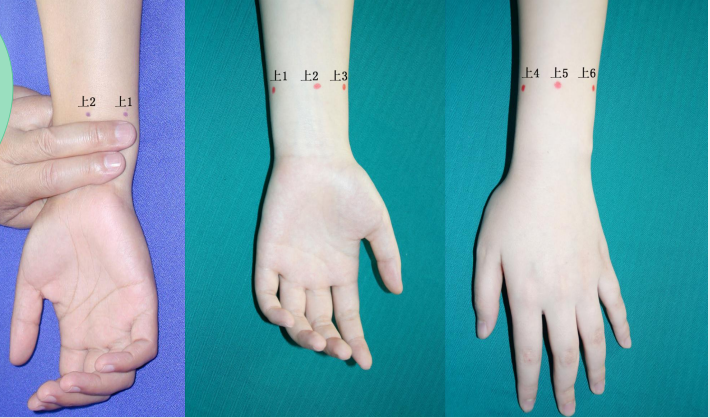


Figure 3

Ankle: arranged in the medial malleolus and lateral malleolus about three horizontal fingers and one circle, recorded as 1~6. As shown in Figure Figure 4


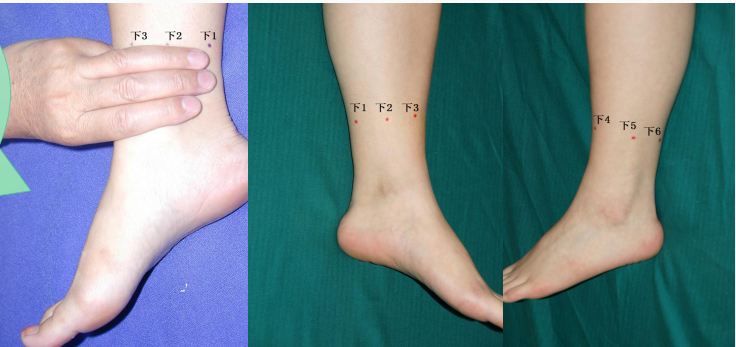


Figure 4

In the process of acupuncture, the position of the needle entry point should be adjusted according to the actual situation of the skin, such as whether there is scar in the local skin, the direction of acupuncture, etc., so that the needle puncture under the skin to reach an appropriate length, that is, the needle entry point is not absolutely fixed.

④ Operation process of the wrist and ankle needle

Before intervention, avoid acupuncture point local blood vessels, determine the constituency, into the needle as the center, with 75% alcohol routine disinfection into the needle around the skin 2 times, wrist ankle needle with 30 degrees into the skin, confirm the needle after the dermis, light twist needle handle, make the needle along the superficial surface slowly into the needle, until the needle body 2mm stop into the needle, and then properly fixed the handle with tape. During the injection, it is appropriate to feel loose without resistance, and the patient does not have any acid, numbness, distension, pain and other special feelings. If the acid, numbness, distension and pain are caused during the puncture, the Angle of the needle should be adjusted according to the patients main complaint, and the needle should be reinjected and fixed.

**2.Name and specification of study medication / device (if involved)**

Using Shukang brand 0.25mmx25mm millineedle, which is 1 inch needle, the manufacturer is Changchun Aikang Medical Equipment Co., LTD.

**3.Consolidated drugs**

Drugs that can be used together: antiviral drugs, non-steroidal analgesic drugs

**VI.**Test **items and test time point**

Uninvolved

**VII.Efficacy evaluation criteria**

1. Treatment effect: refer to the "Guiding Principles for Clinical Research of New Chinese Medicine" promulgated by the Ministry of Health in 1993. Clinical cure: pain loss; effective: 2 points less pain intensity; effective: 1 point less pain intensity; ineffective: less than 1 point less pain intensity. Significant efficiency = significant effect / total cases 100%.

2. Pain score: record the most pain points within 24 hours before the observation point according to the pain visual simulation evaluation method (VAS evaluation method in mm) supervised by the Pain Society of Chinese Medical Association.0 points: painless: 3 points below: slight pain, can endure: 4 points-6 points: the patient pain and affect sleep, still can endure; 7 points-10 points: the patient has gradually intense pain, pain unbearable, affect appetite, affect sleep.

3. Duration of pain (d): the time it takes for the patient to feel the pain until the pain disappears completely.

Items ①, ②, and ③ were recorded before each treatment on Day 1,3,5,7 and on day 28 of pain (calculated as the first day of pain). If item ③ was not recorded on Day 28 of pain (not relieved or disappeared), it was recorded as 28 days. The intensity and apparent efficiency of pain relief in both groups were calculated as the best score of pain relief after treatment.

**VIII.Observation and management of adverse events**

Adverse events, defined as medically induced injury, contrary to the natural outcome of the disease, prolonging the patients length of stay, and all events leading to disability, including preventable and unpreventable adverse events. Unpreventable adverse events refer to unpreventable injuries caused by correct medical behavior; preventable adverse events refer to damage caused by preventable errors or equipment failure in medical treatment.

**1. Subcutaneous bleeding**

Wrist and ankle is more activity, and at the end of the limbs, arteriovenous, rich blood supply, subcutaneous vein network, vascular distribution varies from person to person, subcutaneous fat layer thin thicker vein blood vessels can see, acupuncture can be avoided, but fat layer thick subcutaneous blood vessels is not easy to identify, acupuncture hard to avoid injury and blood vessels, and appear subcutaneous hemorrhage.

**processing method:**

In order to prevent subcutaneous bleeding, try to avoid injuring the thicker vein when entering the needle, and entering the needle should be slow. If the patient feels pain at the wall of the needle and the tip of the needle, try to penetrate the skin at the tip of the needle, remove the needle immediately and stop the bleeding, explain to the patient to eliminate his concerns.

**2. there may be a dizzy needle, resulting in a fall, shock and other phenomena.**

**processing method**:

When the needle occurs, the patient first feel nausea, fatigue, dizziness, or tinnitus, blurred vision, or feel black, pale, cold sweat. With superficial breathing, cyanosis, unconscious, unable to stand, collapsed, and was in shock.

Once dizziness occurs, stop acupuncture immediately, pull out the patient should lie immediately; untie the patients collar, keep breathing unobstructed: pay attention to blood pressure changes, or drink warm water or sugar water, give oxygen if necessary, generally can return to normal within a few minutes.

**IX. Research quality control and quality assurance**

The main factors influencing the study include: selective bias, measurement bias and confounding bias. Due to the particularity of the trial design, subjects cannot be blind, which is not blind, and measurement bias is easy to occur. In order to control the research quality and reduce bias, the randomized control scheme should be strictly followed, the research subjects should be strictly restricted, the inclusion and exclusion criteria should be strictly implemented, and the quality should be strictly controlled during the trial.

**1. use the same batch of micropins**

In the process of intervention, the 0.25mmx25mm millineedle, that is, 1 inch millineedle. The manufacturer is Changchun Aikang Medical Equipment Co., LTD.

**2. Division of labor of researchers and related personnel training**

Members of the research group consisted of trainers, assessors, and intervention personnel. The trainers is responsible for training the knowledge of wrist and ankle needles and puncture techniques, and reviewing the content of the evaluation sheet and the intervention process. The assessors consists of one nursing graduate student and two nurses in charge to evaluate patients with herpes zoster, distribute and collect data. The intervention staff by TCM specialist nurses will intervene on the included study subjects, and during the intervention, operate in strict accordance with the standard operation procedures.

Before the intervention, patients should be trained on wrist and ankle needles, especially on pain-related knowledge, to inform the true reflection of pain degree and the correct expression of pain degree.

**3. Strictly control the quality during the trial: strict randomization:**

the included subjects shall be divided according to the random number table; the data shall be collected by three trained team members to control the trial bias; double entry after data verification.

**X. Data security monitoring**

The clinical study will develop a corresponding data safety monitoring plan according to the risk size. All adverse events are recorded in detail, properly handled and tracked until properly resolved or the condition is stable, and serious adverse events are reported to the ethics committee, competent authorities, sponsor and drug regulatory authorities as required; the principal investigator conducts regular cumulative review of all adverse events and evaluates the risks and benefits of the study if necessary to ensure the safety and rights of subjects. Studies greater than the minimum risk will schedule an independent data supervisor to monitor the study data, and the high risk study will establish an independent data safety monitoring board to monitor the cumulative safety data and effectiveness data to make recommendations for the study.

**XI. Statistical treatment**

Statistical analysis was performed using SPSS22.0, Measurement data meeting the normal distribution are described by the mean ± standard deviation, M (P 25 to P 5), Measurement data consistent with normal distribution and equal variance were analyzed by F test for the differences between the four groups, Compliance to the normal distribution but uneven variance: does not conform to the normal distribution, Differences between the four groups were used by Kruskal-Wallis rank sum test, And pairwise comparison differences: within group before and after treatment and consistent with normal distribution using paired design two independent samples · test, Unconforming normal distribution was determined using two independent sample rank sum tests, P <0.05 was considered to be statistically significant.

**XII. Ethics principles and requirements for clinical research**

Clinical studies will follow the Declaration of Helsinki of the World Medical Congress, the National Health Commissions Measures for the Ethical Review of Biomedical Research Involving People, and the Administrative Measures for Investigator-initiated Clinical Studies by Medical and Health Institutions. Before the start of the study, the ethics committee will approve the test protocol, and the information about the clinical study shall be recorded in the medical research Registration and Filing Information System only after officially starting for 30 days in advance.

Before each subject is enrolled, the investigator has the responsibility to fully and comprehensively introduce to the subject or / or their legal guardian the purpose, procedures and possible risks of the study and sign a written informed consent that they may refuse to participate in the study or withdraw at any time at any stage of the trial. They will not be unfairly treated and will not affect the relationship with the clinician / nurse or their normal treatment. The informed consent form should be retained as a clinical study document for future reference. Personal privacy and data confidentiality will be protected during the study.

**XIII. Research progress**

| start time | terminal time | Stage goal |
| --- | --- | --- |
| 2023-01-01 | 2024-6-30 | Start to enroll patients, treat and collect relevant clinical data, and enroll all cases. |
| 2024-07-01 | 2024-9-30 | Summarize the data collected in the previous year, and analyze them by using statistical software. |
| 2024-10-01 | 2024-12-31 | Summarize the research results and complete the conclusion. |

**XIV. Participants**

| surname and personal name | professional ranks and titles | specialty | assignment | GCP Training (time) |
| --- | --- | --- | --- | --- |
| Du xiaobo | professional | Oncology | Project design, supervision, and paper revision | Yes(2020) |
| Li generation wen | nurse-in-charge | Traditional Chinese medicine nursing | Project design, statistics, and paper writing | not have |
| Deng Lanlan | nurse-in-charge | nursing supervision | technological guidance | not have |
| Luo Xia | nurse-in-charge | nursing supervision | Data collection and input, statistics ,and paper writing | not have |
| Tan Dongmei | physician | Traditional Chinese medicine skin | Project implementation and data collection | not have |
| He yuanli | physician | Skin clinical | Project implementation and data collection | not have |
| Wang Juan | nurse-in-charge | Traditional Chinese medicine nursing | Project implementation, data collection, and paper writing | not have |
| Li Yanxia | nurse-in-charge | nursing | Project implementation, data collection, and paper writing | not have |
| Zhao Xiankun | physician | Traditional Chinese medicine clinical | Project implementation, data collection, and paper writing | not have |

**XV. Main references**

[1] Li Juan, Wu Jiang. Epidemiology and vaccine immunization strategies for herpes zoster [J]. The Journal of Chronic Diseases, 2021,22(08):1145-1151.DOI: 10.

16440/J.CNKI.1674-8166.2021.08.01.

[2] Marra F, Parhar K, Huang B, et al. Risk factors for herpes zoster infection: a meta- analysis [J]. Open Forum Infect Dis, 2020, 7(1):ofaa005.

[3] Torcel- Pagnon L, Bricout H, Bertrand I, et al. Impact of underlying conditions on zoster- related pain and onquality of life following zoster [J]. J Gerontol A Biol Sci Med Sci, 2017, 72(8):1091-1097

[4] Wei Min, Yan Yan. Progress in the pharmacological treatment of herpes zoster [J]. Journal of Clinical Pharmacotherapy, 2019,17 (11): 33-37.

[5] Wang Yanqing, Meng Xianghui. Progress in the application of corticosteroids in herpes zoster [J]. Journal of Mudanjiang Medical College, 2021,42(02):141-143.DOI:10.13799/j.cnki.mdjyxyxb. 2021.02.037.

[6] Jining, Zhao Xing, Zeng Xin, Chen Changming. Progress in nucleoside antiherpesvirus drugs [J]. International Journal of Oral Medicine, 2018,45 (03): 351-357.

[7] Yi Weijun, Wang Zhenzhen, Zhang Lin, Li Li. Semiconductor laser treatment of neuralgia after herpes zoster [J]. Laser Magazine, 2020,41(11):203-205.DOI:10.14016/j.cnki.jgzz. 2020.11.203.

8] Duan Yi wen, Guo Shuping. Progress in postherpetic neuralgia [J]. Chinese Journal of Multiple Organ Diseases in the Elderly, 2019,18 (07): 552-556.

[9] Fan Ruiqiang. acute posterior ganglionitis [M]. Beijing: China Press of Traditional Chinese Medicine, 2012:16-17.

[10] Li Qian, Wu Mingxia. Overview of the treatment of herpes zoster by traditional Chinese medicine [J]. Chinese Medical Guide, 2020,22 (12): 865-869..

[11] Zhuang Ting, Liu Xuemei, Wang Yaping, Long Yajing, Tang Rong, Li Simeng, Wang Wei. Clinical study of Huangdi internal acupuncture combined with aciclovir [J / OL]. Clinical research of traditional Chinese medicine: 1-3 [2022-09-18]. https://er.szlib.org.cn:443/rwt/331/http/NNYHGLUDN3WXTLUPMW4A

/ kcms/detail/11.5895.R.20220815.1013.006.html

[12] Wang Nana, Wang Wei, Wang Haiquan. Overview of TCM external treatment of postherpetic neuralgia [J]. Practical Journal of Traditional Chinese Medicine, 2021,37 (01): 154-155.

[13] Chen Qing. Clinical observation of bloodletting therapy for herpes zoster related pain [D]. Hubei University of Traditional Chinese Medicine, 2022.DOI:

10.27134/d.cnki.ghbzc.2022.000374.

[14] Huang Gaozhen, Wang Liang, Wu Jingxuan, Wu Bo, Deng Jie, Huang Xiaojun. Comparison of the efficacy of traditional Chinese medicine, acupuncture and western medicine for antiviral treatment of herpes zoster [J]. Journal of Hubei University for Nationalities (Medical edition), 2021,38(04):16-20.DOI:10.13501/j.cnki.42-1590/r. 2021.04. 004.

[15] Yang Ke, Du Yuzhu, Shi Jing, Wang Jianling, Sun Yanhui, Xing Haijiao, Li Xiaofeng, Xu Jing, Zhang Xuanping, Zhang Xin, and Jia Chunsheng. To explore the dominant diseases and clinical application characteristics of wrist and ankle needle therapy by using data mining technology [J]. Chinese acupuncture, 2019,39(06):673-678.DOI:10.13703/j.0255-2930.2019.06.029

[16] LAN Lei, Zhang Guoxin Shan. Wrist / ankle needle therapy [M]. Version 2. Beijing: China Medical Science and Technology Press, 2012.

[17] Xu Yunxiang, Chen Guizhen. Efficacy observation of different needle retention time on ankle soft tissue injury [J]. Acupuncture in China, 2001 (10): 33-34.

[18] Guo Jenny, Chen Zhiyao, Xu Jingxin, Peng Dezhong. Analysis of the clinical application of wrist and ankle needles in recent 5 years [J]. Massage and Rehabilitation Medicine, 2020,11(08):56-59.DOI:10.19787/j.issn.1008-1879.2020.08.021.

[19] MAO Guanglan, Qi Guohao, Zhang Xinying, et al. Observation of acupuncture in the treatment of postherpetic neuralgia [J]. Journal of Xinxiang Medical College, 2012,29 (7): 514-516.

[20] Fan Ling, Zhang Ling, Zeng Lingchuan. Clinical observation of prolonged needles and collaterals in the treatment of herpes zoster neuralgia [J]. Sichuan Traditional Chinese Medicine, 2006,24 (5), 107-108.
